# Pediatric Bowel Management Options and Organizational Aspects

**DOI:** 10.3390/children10040633

**Published:** 2023-03-28

**Authors:** Elizaveta Bokova, Wendy Jo Svetanoff, Marc Aaron Levitt, Rebecca Maria Rentea

**Affiliations:** 1 Department of Surgery, Children’s Mercy Hospital, Kansas City, MO 64108, USA; eobokova@gmail.com; 2 Comprehensive Colorectal Center, Department of Surgery, Children’s Mercy Hospital, Kansas City, MO 64108, USA; 3 Division of Colorectal and Pelvic Reconstruction, Children’s National Medical Center, Washington, DC 20001, USA; 4 Department of Surgery, University of Missouri-Kansas City, Kansas City, MO 64108, USA

**Keywords:** bowel management, antegrade flushes, antegrade continence enema, Malone, cecostomy, bootcamps, outcomes, education, collaborative, telemedicine

## Abstract

A bowel management program (BMP) to treat fecal incontinence and severe constipation is utilized for patients with anorectal malformations, Hirschsprung disease, spinal anomalies, and functional constipation, decreasing the rate of emergency department visits, and hospital admissions. This review is part of a manuscript series and focuses on updates in the use of antegrade flushes for bowel management, as well as organizational aspects, collaborative approach, telemedicine, the importance of family education, and one-year outcomes of the bowel management program. Implementation of a multidisciplinary program involving physicians, nurses, advanced practice providers, coordinators, psychologists, and social workers leads to rapid center growth and enhances surgical referrals. Education of the families is crucial for postoperative outcomes, prevention, and early detection of complications, especially Hirschsprung-associated enterocolitis. Telemedicine can be proposed to patients with a defined anatomy and is associated with high parent satisfaction and decreased patient stress in comparison to in-person visits. The BMP has proved to be effective in all groups of colorectal patients at a 1- and 2-year follow-up with social continence achieved in 70–72% and 78% of patients, respectively, and an improvement in the patients’ quality of life. A transitional care to adult program is essential to maintain the same quality of care, and continuity of care and to achieve desired long-term outcomes as the patient reaches adult age.

## 1. Introduction

Bowel management is a one-week program with a multidisciplinary care team developed to establish an optimal regimen for treating constipation and fecal incontinence. Until a successful and sustainable regimen is defined, the evacuation of stool is monitored with frequent abdominal radiographs to achieve sufficient colonic emptying. Successful bowel management decreases emergency department visits, hospital admissions, and improves the quality of life in this population [1,2,3].

A multidisciplinary, individualized approach is the key to successful management as age, functional status, behavior, and underlying diagnosis contribute to the complexity of care for these children. The elements of a structured bowel management program (BMP) include a collaborative approach between colorectal surgeons, urologists, gynecologists, gastrointestinal specialists, neurosurgeons, and orthopedic surgeons to manage patients with associated anomalies [4].

Until recently, only early BMP outcomes were mentioned in the literature [5,6]. Now large studies of one-year results of a structured BMP have been completed [3,7]. Our goal is to report updates in the bowel management program in a series of manuscripts covering BMP considerations for patients with anorectal malformations (ARMs), Hirschsprung disease (HD), spinal anomalies, and functional constipation (FC). The current article focuses on the use of antegrade continence enemas (ACEs) when a mechanical emptying is needed, and organizational aspects of a dedicated BMP, such as the vital collaborative approach, family education, implementation of telemedical technologies, and intermediate- and long-term outcomes of a structured BMP.

## 2. Methods

A search was made of literature published before March 2023 in Medline/PubMed, Google Scholar, Cochrane, and EMBASE databases, including original studies, meta-analyses, randomized controlled trials, and systematic reviews, focusing on manuscripts and books published over the last 5–10 years. Search keywords included: “bowel management”, “enema”, “antegrade continence enema”, “Malone”, “cecostomy”, “outcomes”, “education”, “teaching”, “collaboration”, “telemedicine”, and “transition of care”. Ninety-three of the selected articles were included in the current review.

## 3. Antegrade Continence Enemas

Constipation is a widespread problem in the pediatric population affecting up to 30% of children [8], with the need for ED visits in 15% of cases [9]. More than 90% of patients can be successfully managed medically. However, a group of patients “fail” medical management. There is no clear definition of “failure” of medical management that lead to heterogenic data in the literature [10]. Patients with an ARM, HD, a spinal anomaly, or FC that do not respond to laxatives, stool softeners, rectal enemas, and/or non-medical treatment or cannot tolerate rectal enemas daily, are candidates for antegrade flushes [11]. Antegrade continence enemas (ACEs) are more comfortable for patients in comparison to rectal administration and allow for more independence. It is considered a medical treatment, as, like a rectal enema; it is the flush that works to empty the colon, not the route of administration.

### 3.1. Definition and Indications

ACE is performed when the appendix (appendicostomy) [12], a cecal flap (neoappendicostomy) [13], or the cecum itself (cecostomy) [14] is brought to the level of the skin. Patients who have failed medical management with rectal enemas and laxatives are candidates for an ACE procedure. Flushes are administered through the connection between the bowel and the skin in an antegrade manner, to mechanically empty the colon at a reliable time, and to achieve social continence.

There is a variety of protocols for ACEs administration among physicians [11]. The assessment of outcomes is complicated due to the different measures of “success” used by various authors [11]. The timing of this procedure occurs either when the patient wants to become more independent with their bowel regimen [10], cannot tolerate rectal therapies, or fails medical treatment with stimulant laxatives and/or water-soluble fiber after having undergone a bowel management program [15]. Some 44% of physicians perform an ACE procedure on achievement of the toilet training age, at a minimum age of 4 years [11]. The recipe used for successful rectal enemas often translates to be given antegrade via an ACE. The role of antegrade flushes as a bowel management option in patients with an ARM, HD, spinal anomaly, or FC is described in the related article series “State of the Art Bowel Management for Pediatric Colorectal Problems”.

### 3.2. Appendicostomy vs. Cecostomy

Two systematic reviews and a meta-analysis have been completed on Malone appendicostomy and cecostomy. Mohamed et al. assessed 2096 patients in 40 studies and revealed that the overall complications rate was higher in patients that underwent an appendicostomy (42% vs. 17%) [16]. The risk of the need for surgical revision was also higher in the appendicostomy groups. Patient satisfaction, quality of life improvement, and continence rates were reported to be similar in both groups of patients [16,17]. However, Halleran et al. reported that a cecostomy had less promising results as compared to a Malone appendicostomy in regard to leakage (22% vs. 3%), wound infections (28% vs. 7%), and the need for additional interventions (35%) for inability to flush, tube dislodgement, or major complications such as intraperitoneal spillage and tube misplacement in the ileum [18]. Cecostomy has also been shown to be associated with a higher rate of postoperative abdominal pain (54% vs. 14%) [19].

### 3.3. Intraoperative Decision-Making

Intraoperatively, the decision about the ACE type to be performed is made according to the: (1) length of the appendix, (2) vascular supply, and (3) lumen of the channel that could allow for future catheterizations. A Malone can be performed if the appendix is at least 4 cm in length, is vascularized with at least one mesenteric vessel, and is wide enough to pass an 8–10 Fr catheter. Otherwise, if a channel is required, a neoappendicostomy can be created.

**Figure 1:**
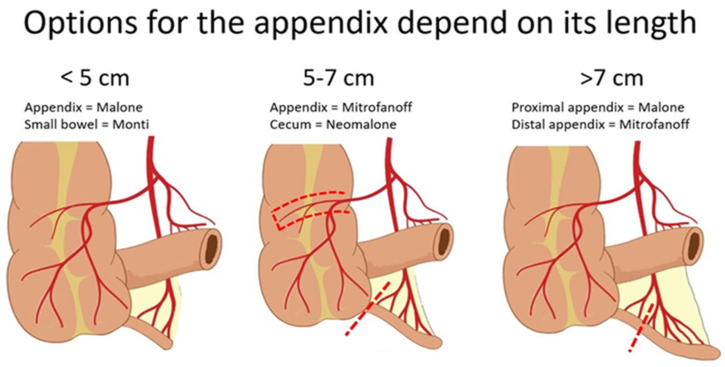
Decision-making in terms of access for antegrade flushes and urinary diversion. If the appendix is shorter than 5 cm, a Malone is created, and urinary access is ensured by creating a Monti channel. If the appendix is 5–7 cm long, it is used for a Mitrofanoff, and a Neomalone is created for antegrade flushes of the colon. An appendix longer than 7 cm allows for a split appendix procedure. Reprinted from Halleran DR, Sloots CEJ, Fuller MK, Diefenbach K. Adjuncts to bowel management for fecal incontinence and constipation, the role of surgery; appendicostomy, cecostomy, neoappendicostomy, and colonic resection. Semin Pediatr Surg. 2020 Dec; 29(6): 150998, p. 4, with permission from Elsevier.

In multidisciplinary centers, preoperative planning is done in collaboration with urologists, which considers the needs of both colorectal and urology teams. Some patients with an ARM or spina bifida who need antegrade access for colonic emptying have associated urologic anomalies [20,21] and might require both antegrade flushes for bowel management and a Mitrofanoff channel for urinary access or ease of bladder emptying. From the urologic standpoint, the appendix may be needed for a Mitrofanoff and is the best conduit, superior to a Monti ileovesicostomy (made from small bowel), for managing urinary incontinence. In certain cases, the appendix can be shared using a split appendix technique, but this depends on the length of the appendix with sufficient vascularization and lumen width [22,23] (Figure 1). However, a split technique has been found to have a 47% complication rate and a more than twice higher risk of the need for future revisions, mostly due to isolated stomal stenosis (65%), in comparison with an isolated Mitrofanoff or Monti channel [24].

### 3.4. Postoperative Care

The caregivers are taught to perform the flushes for the first month after Malone/Neomalone appendicostomy if a temporary catheter such as a feeding tube or Foley catheter has been utilized. For children with a primary button such as MiniACE™, the first exchange takes place at 3-month intervals—effectively providing a low-profile option for children who require more time to learn catheterization of the channel [25,26]. MiniACE™ results in more granulation tissue [25], and 12% of patients require revisions for prolapse of the appendicostomy [26]. However, for those who have multiple catheterizable channels to learn about or have behavioral barriers, this tube is helpful as a bridge to delayed mastery of channel catheterization.

### 3.5. Outcomes

Some studies have shown that patients can “learn” to control their passage of stool with the antegrade flushes and, at that point, do not need their ACE anymore. The mechanism of ACE action is the administration of enemas in an antegrade manner. The physiological pathway of the solution simulates the stool, and the patients can train to sense that the flush is coming and to control the stool passage with their anal sphincters to improve their bowel control. Over time, this mechanism of bowel control gets trained enough to detect and hold the stool, the ACE serves as a bridge to continence. Combined with improving constipation, it reduces the need for further ACE administration. Dolejs et al. reported 43% of patients were able to become independent from ACE flush with at least 24-month follow-ups [27]. Others had less promising outcomes, with 74% of patients still using their ACEs 5 years after the ACE procedure [28]. Rodriguez et al. reported that in 64% of patients with constipation, the frequency of antegrade flushes could be decreased, while 28% successfully discontinued ACE administration at a 2-year (14–74 months) follow-up [11].

Antegrade flushes greatly impact the quality of life (QoL). Brophy et al. studied QoL following laparoscopic cecostomy tube placement. They reported that antegrade enemas led to a significant improvement in stool-related concerns and overall QoL 3 months after the procedure, with an increasing rate until the last follow-up date. Interestingly, there was no statistically significant correlation between the QoL improvement and the initial diagnosis that led to the ACE placement [29].

## 4. Long-Term Collaboration and Bowel Management Program

### 4.1. Collaborative Approach

Management of patients with an ARM, HD, spinal anomaly, and FC requires care from multiple pediatric specialists, particularly as care extends beyond the surgical reconstruction throughout the patient’s childhood. The long-term effects of ARMs and the associated comorbid conditions, bowel, bladder, and reproductive issues create a long-term public health burden causing reduced independence, decreased quality of life, increased healthcare utilization needs, and reduced life expectancy. The benefits of a multidisciplinary center include decreased inpatient hospitalization, clinic visits, adverse anesthetic events, and intubations, and improved transition of care into adulthood [1,30,31,32,33].

For many children who visit multidisciplinary centers, such as those with an ARM, 67% enroll in dedicated programs to manage incontinence [34]. The first report describing a single-institution experience of organizing care in a multidisciplinary unit to treat colorectal diseases included 1258 patients from 63 countries, 514 of whom participated in a bowel management program. The collaborative approach in the decision-making process regarding management strategy facilitated treatment and resulted in better outcomes [31]. Implementing a multidisciplinary program led to rapid center growth within one year [33].

It is important to note that physicians, nurses, advanced practice providers, coordinators, psychologists, and social workers are important components of a collaborative center [31]. Nurses ensure coordination among physicians and are responsible for family education, which is crucial for achieving the best outcomes [33].

### 4.2. Importance of Family Education

The caregivers play an important role in the BMP outcomes by caring for their children daily. The treatment prescribed in BMP can be time intensive; for instance, antegrade flushes require time for their administration and can affect the daily routine of both patients and caregivers, and therefore negatively affect their QoL [7]. This can lead to poor treatment compliance resulting in more hospital admissions, morbidity, and mortality rates [7,35].

Family health literacy is critically important for long-term care outcomes in colorectal patients, even though its importance might be difficult to assess objectively. Dingemans et al. studied the health literacy of 127 US and Honduran caregivers whose children underwent an ARM reconstruction. Interestingly, despite a low health literacy rate in Honduras, the impact of the health-related QoL (HRQoL) between the two populations was not significantly affected. This could be a bias due to the pressure the caregivers could experience trying to give socially desired answers [36].

Despite the data inconsistency, there is no doubt that in most cases, the caregivers are the first ones to be able to reveal the symptoms of failed management or complications. This is especially critical in patients with HD. Education of these families allows for detecting the signs of Hirschsprung-associated enterocolitis (HAEC) and, if present, initiating the performance of rectal irrigations, given that supplies and instructions have been provided [37].

### 4.3. Telemedicine

A dedicated BMP experience is vital for many children to achieve continence with their anatomy and improve QoL. Colorectal patients who lack local specialized care can benefit from telemedical technologies, allowing the families to receive medical care in a tertiary center without traveling long distances [38]. The importance of applying remote technologies was especially evident during the COVID pandemic [39]. Institutions report up to 72% of patients undergoing BMP remotely with improved continence after the visits and decreased rate of stress for the patients and their families compared to in-person visits [39]. A single institution study showed that among colorectal patients after their reconstruction, 44% of families (all of which had children with severe malformations) preferred in-person visits to telephone calls [40]. This might result from the absence of face-to-face contact during a telephone consultation that could be ensured using video technologies.

Another recent study showed that half of the responders agreed to a surgical visit via telemedicine for their children, with a higher agreement rate in those who underwent surgery without complications. Of the respondents that agreed to an in-person visit as the only option, 63% were against telemedicine, 35% were concerned about the competence of the physician, and the others reported concerns about cost, privacy, and lack of infrastructure at the hospital to support telemedicine. Further education on surgical telementoring is required to improve the attitude of families toward telemedicine [38].

Not all patients can be managed remotely. Children with defined anatomy can benefit from remote management whereas others require in-person assessment of the anatomy through examination under anesthesia, cystoscopy, vaginoscopy, contrast study, or manometry not to miss anatomic defects and to indicate optimal treatment. After the anatomy is clearly defined, remote management can be offered. Without this assessment, anatomic causes of their continence and soiling, such as anal stenosis, can be missed, leading to suboptimal results for telemedicine technologies [41].

## 5. Bowel Management Boot Camps

### 5.1. Assessment of Quality of Life and Fecal Continence Scoring

A standardized protocol should be used to reveal the program outcomes when applying any novel technique or program. BMP directly effects fecal constipation and incontinence, highly impacts the social development of the patients, and decreases stress experienced by caregivers [2]. Regarding standardization of colorectal care and follow-up, specific score systems were developed to measure the QoL and severity of functional disorders. There are several protocols utilized to assess continence (Baylor Continence Scale), the severity of constipation (Cleveland Constipation Scoring System), and soiling (Cincinnati Fecal Incontinence Scale) [3,39]. The quality of life is mostly assessed using the Pediatric Quality of Life Inventory (PedsQL), considering functional results, symptom improvement, and social adaptation [42]. In patients with fecal incontinence, the Cincinnati Fecal Incontinence Scale (CINCY-FIS) is highly sensitive to QoL measurement, including patient changes and parental stress [43]. Dysfunctional elimination syndrome or bladder bowel dysfunction is scaled based on the Vancouver Symptom score [44].

### 5.2. Outcomes of the Bowel Management Program

Until recently, only short-term outcomes of BMP were reported with a success rate of up to 95% at one week [5,6]. Clearly maintaining success is a challenge. In 2019, Kilpatrick et al. published initial BMP week results and long-term outcomes of severe constipation and fecal incontinence treatment. The review of 285 patients showed that at the end of the BMP week, 87% of patients (with 6% non-adherent) had no accidents and less than two smears per week which was defined as successful management (social continence). Social continence was achieved in 72% and 78% of cases at a follow-up of one and two years, respectively [45].

One-year outcomes after completing a dedicated bowel management program are important indicators to understand its sustained positive impact on continence and quality of life. A single institution review by Wood et al. evaluated one-year outcomes of 222 patients with ARMs referred for soiling and managed with rectal/antegrade enemas (67%) or oral laxatives (37%) after enrollment in the bowel management program. One year after the program completion, 150 (70%) patients were clean on rectal/antegrade enemas (73%) or laxatives (27%). There was also a significant improvement in the Baylor and Vancouver scores, total PedsQL, and PedsQL HRQoL physical function and psychosocial domain. A structured BMP was also shown to improve urinary incontinence in patients with soiling [3].

Similarly, a single institution prospective cohort study included 342 patients with fecal incontinence assessed using the CINCI-FIS. Some 63% of patients were managed with rectal or antegrade enemas, and 37% were on a laxative trial. Fecal incontinence improved within 2 weeks and was sustained for a year, as well as the quality of life for the patients and their caregivers. Patients with congenital diagnoses had lower fecal incontinence scores and a lower rate of CINCI-FIS improvement compared to patients with diseases diagnosed later in life. Importantly, one year following BMP, 20% of patients had returned to daily daytime involuntary bowel movements, and 15% reported daily nighttime soiling [2]. For further information on BMP protocols and outcomes in each group of colorectal patients, see the related manuscript series “State of the Art Bowel Management for Pediatric Colorectal Problems”.

## 6. Transition of Care

In the United States, 14% have unique healthcare needs with one in five households having a child with special care requirements [46]. Of the patients reaching the age of 21 years, 45% need access to a physician aware of their disease and qualified to provide sufficient management. In 2010, a joint commission of the American Academy of Pediatrics, American Academy of Family Physicians, and American College of Physicians stated the importance of the organization of the transition from pediatric to adult specialties for patients with unique healthcare needs [47]. Functional outcomes and quality of life may deteriorate as the patient grows. Therefore, a formal pathway of care transition from the pediatric to an adult setting is required once the patient reaches adult age [48]. Over the past ten years, more manuscripts have been published on the application of the transition program and long-term outcomes in patients with colorectal diseases [49,50,51].

### 6.1. Indications for Transition

Children with colorectal diseases have unique long-term healthcare needs that extend into their adulthood [52]. Patients with a history of surgery for a colorectal disease are in a significantly higher need for transition to adult specialties compared to other pediatric surgery patients [53]. The lack of a structured transition program for patients and families negatively affects their long-term prognosis [54], leaving them without dedicated healthcare and making them seek continuous management by pediatric specialties [55].

The challenges colorectal patients may face in adulthood include psychological issues, bladder dysfunction, and sexual concerns exacerbated by the lack of transitional care [54,56,57,58,59,60,61,62,63,64,65]. Among stooling concerns, there is a high prevalence of bowel management concerns, including fecal incontinence (32–85%) and constipation (50%) in young adults with colorectal conditions transited to adult care [51,66]. However, surgical complications remain the predominant reason for seeking transitional care [55]. These statistics may change as the result of surgical techniques performed has improved [55]. Specifically, in 1990 the surgical technique of PSARP for patients with ARMs became incorporated into pediatric surgical practice and training. Acker et al. compared ARM patients that underwent a primary reconstruction before PSARP was described to those who were operated on after 1990 using PSARP. The first group of patients sought transitional care mostly for a redo surgery (59%). Since the PSARP technique became widespread, additional surgical needs, such as a Malone appendicostomy and rectal prolapse repair (40% and 20% respectively), have been the most common reason for transition [55].

Given the improvement in reconstruction, the focus of transitional care will most likely shift over the upcoming decade from the treatment of postoperative complications to bowel management. Rectal prolapse, irregular menses, tampon difficulty, dysmenorrhea, and urinary incontinence are also becoming more prevalent reasons for adult care [55].

### 6.2. Multidisciplinary Transition

Pediatric surgical subspecialties have developed widely and significantly improved the quality of pediatric surgical care. A continuous long-term multidisciplinary approach is required for patients with colorectal diseases and transition to a collaborative center including care for urologic, gynecologic, gastroenterologic, and sexual concerns is required to allow for the same quality of care the patients received in childhood [51,52,67,68] and achieve optimal outcomes [55,69]. Sexual and fertility concerns should also be addressed [70] with the current rate as low as 11% of adult patients with ARM or HD involved in sexology or andrology care [69]. Those with ARMs require management of associated VACTERL malformations if present [71].

Bladder dysfunction is common in adults with colorectal conditions [72]. They can experience recurrent urinary tract infections (34–70%), urinary incontinence (46–52%), CKD (20%), and other urologic concerns [66,73]. Urologic care transition was shown to decrease the use of emergency services [74]; however, it is currently associated with a lower satisfaction rate when compared to pediatric urology care [75], confirming the need for further improvements.

Gynecologic issues can also affect colorectal patients, especially those with anorectal malformations [68,76,77]. Family planning and obstetrical concerns addressed individually with the discussion of both risks and benefits and shared decision-making process [76,77] could improve the quality of life in these patients [68].

### 6.3. The Right Time for Transition

Of pediatric surgeons, 33% discharge patients with an ARM from their practice at less than 10 years of age [78], while after reaching this age, 77–87% struggle with fecal incontinence and/or constipation [56]. Urologic and sexual concerns are also common after the first decade of life with up to 31% of patients having urinary continence, 41% presenting with ejaculation dysfunction, and 12% having erectile dysfunction [56].

The recommended time for transition start is 12–13 years of age with its completion at 18–21 years [47,79,80,81,82]. Some institutions suggest a later age to start the transition [67,78]; however, an earlier initiation of the process gives the family and the patient more time to prepare for the upcoming changes in their healthcare [81,83,84]. It is important to keep in mind that the traditional transition time of 18 to 21 years is coincident with other significant life events which can cause additional stress for the patient and their family.

### 6.4. Readiness for Transition

The transition should have a standardized structure to ensure that patients switch from pediatric to adult healthcare providers without interruptions. To allow for a coordinated transition, it is crucial to ensure that healthcare providers, patients, and their families are prepared for the transition of care [47,85].

#### 6.4.1. Providers

To standardize the transition of care, the home institution providers must undergo training and technical support to implement the program, assess families and patients for transition readiness, and develop referral protocols with adult care specialists [47]. Of pediatric surgeons managing patients with colorectal diseases, 68% are concerned about their transition to adult specialties [86]. Surgeons managing exclusively adults are treating a growing number of patients with a history of major reconstructive surgeries during childhood; however, most of them have limited training in pediatric surgery and, therefore, insufficient awareness of the medical issues, particular of these adults, to provide the same quality of care as the patient had in childhood and adolescence. These issues and the detailed plan of care should be discussed with the adult specialties when planning the transition of care.

Botelho et al. conducted a study to assess the care of pediatric surgery patients after reaching 18 years of age. Out of 106 pediatric surgeons, 48% referred patients to an adult surgeon, whereas the rest of the respondents continued to follow these patients in adulthood due to patient preferences, long-lasting physician-patient relationships, or limited knowledge of pediatric surgical diseases among adult surgeons (36%, 28%, and 23% respectively) [86]. Most often, the patients are referred to adult specialties of the same hospital, health insurance network providers, or specialists who the pediatric surgeon trusts [86].

#### 6.4.2. Patient

**Figure 2:**
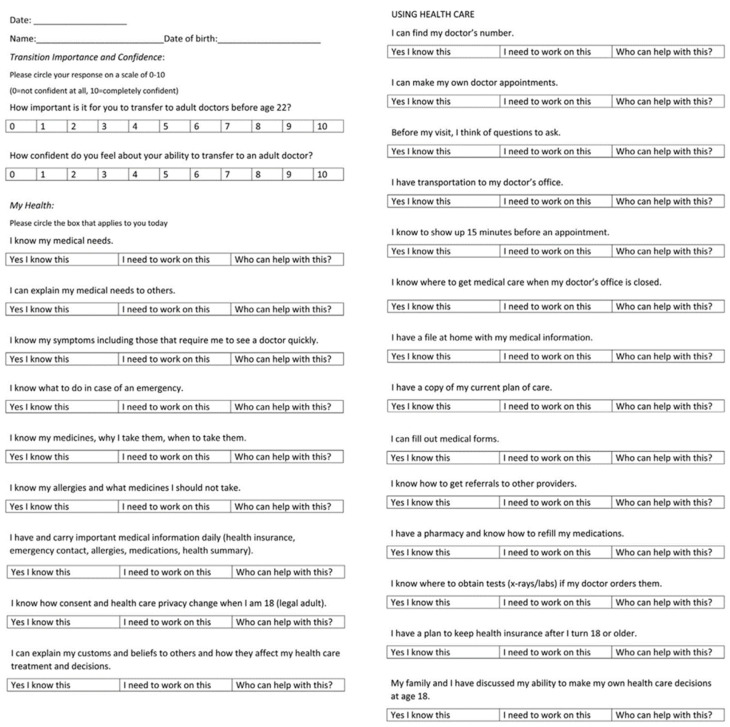
Questionnaire to access the patient’s readiness for the transition of care. Reprinted from Gasior A, Midrio P, Aminoff D, Stanton M. Ongoing care for the patient with an anorectal malformation; transitioning to adulthood. Semin Pediatr Surg. 2020; 29(6): 150991, p. 2, with permission from Elsevier.

It is important to address the fact that the patient becomes the primary decision-maker once transited to adult care. Maximizing their independence and self-determination is crucial for the patient’s ability to take a life-long responsibility for their health and gain control over further management of their condition [47]. Health literacy is an important factor that increases the patient’s understanding of their disease, self-management, and, therefore, readiness for transition, which may be a valuable tool for assessment prior to referral to adult care [87,88,89]. To access a patient’s readiness for the transition of care, standardized questionnaires have been developed (Figure 2) [90].

#### 6.4.3. Family

Change from a familiar pediatric care unit to a new adult center may be stressful for caregivers of patients with unique colorectal needs. In order to allow for smooth transitional care, the concerns of the families should be addressed when planning the transition. Importantly, the new roles of the patient and the family should be discussed and clearly defined, with attention paid to increased responsibility for further care being transferred to the patient once they reach adult age. The family should be engaged in the transition program, with the decision-making now being on the part of the patient [47].

### 6.5. Components of the Transition Program

There are three main components required for a structured transition of care: (1) an officially written transition program, (2) a coordinator of the transition process, and (3) handout materials provided to patients when transitioning to adult specialties [69]. Violani et al. reported that this system is currently far from being implemented in practice with 6/80 (8%) healthcare providers meeting the criteria of an organized transition program. The need for a clear structure of a transition program was reflected in the article by Fernandex et al. that revealed gaps in the information provided to patients arriving at adulthood, including birth control, pregnancy, the health of future children, drug abuse, and other concerns being raised when achieving adult age [91].

### 6.6. Keys to Success and Barriers to Transition

Van der Bent et al. reported that of ARM patients who switched from pediatric to adult care, only 33% had been supported before and during the transition using discussion or instructions, and 29% had been recommended adult providers for referral [92]. The key components determining transition success were knowledge about the disease, well-established communication between healthcare providers, patients, and families, and easy access to adult care specialties [89].

The transition of care is developing and requires further improvement [55,86]. More than 60% of patients are affected by challenges when transitioning to the adult care system. With age, the patients get less adherent to the treatment and have limited access to healthcare services which negatively affects the outcomes [52,93], especially in underserved areas [94]. Lack of expertise of adult physicians in the management of “pediatric” colorectal diseases and associated conditions such as sexual and reproductive dysfunction, poor communication between healthcare providers, family-related concerns (lack of patient’s independence, poor family support), and need for travel outside the geographic area were described as the main causes of distress associated with the transition of care [52].

## 7. Conclusions

Bowel management is a dedicated program with a collaborative approach that allows the patients to be treated for all associated diseases and leads to rapid center growth. The involvement of nurses, advanced practice providers, psychologists, social workers, and coordinators is crucial for a specialized colorectal center. Family education allows the achievement of optimal outcomes and an early start to the treatment of complications, such as HAEC. Telemedical technologies decrease the patient’s stress and are associated with a high satisfaction rate among caregivers. Only patients with defined anatomy should be managed remotely. The one- and two-year outcomes of a bowel management program are promising with 70–78% of colorectal patients achieving social continence. Long-term outcomes depend on standardized multidisciplinary transitional care to adult specialties allowing for the same quality of care as the patient reaches adulthood.

## Data Availability

No new data were created or analyzed in this study. Data sharing is not applicable to this article.
